# The impact of the COVID-19 pandemic on the incidence of herpes zoster

**DOI:** 10.1093/eurpub/ckac131.204

**Published:** 2022-10-25

**Authors:** N Lecrenier, R Parikh, C Wang, D Curran, R Widenmaier

**Affiliations:** 1 Global Medical Affairs, GlaxoSmithKline, Wavre, Belgium; 2 Epidemiology, GlaxoSmithKline, Rockville, USA; 3 Value Evidence, GlaxoSmithKline, Wavre, Belgium; 4 Global Medical Affairs, GlaxoSmithKline, Rockville, USA

## Abstract

**Background:**

There have been several case reports of herpes zoster (HZ) following COVID-19 disease and vaccination. We conducted a non-systematic literature search to elucidate the global effects of the COVID-19 pandemic on the incidence of HZ.

**Methods:**

The literature search was performed in October 2021 using PubMed and Embase. The search string was herpes zoster AND COVID-19. Publications were manually reviewed; case reports were removed.

**Results:**

Three retrospective studies reported the risk of HZ following COVID-19 disease. One study (Bhavsar, 2021) used two US databases and found higher risk of HZ following COVID-19 disease (relative risk [RR]=1.15) and COVID-19 hospitalisation (RR = 1.21), respectively. A strong association between HZ and COVID-19 disease (RR = 5.27) was also reported in a study of the University of Florida patient registry (Katz, 2021). The third study (Barda, 2021) reported no association between COVID-19 disease and risk of HZ (RR = 0.82). In two of the three observational studies in Israel (Furer, 2021 and Barda, 2021), the incidence of HZ was increased following COVID-19 vaccination. The third study (Shasha, 2021) found no association (RR = 1.07). Other studies included a report in Brazil (Maia, 2021) that demonstrated a 35% increase in HZ diagnoses during the pandemic versus pre-pandemic and a published model (La, 2021) that estimated the declining uptake of recombinant zoster vaccine in the US may result in 63,117 avoidable HZ cases in those who remain unvaccinated in 2021.

**Conclusions:**

Emerging data suggest that the COVID-19 pandemic may have increased the risk of HZ and negatively impacted HZ vaccine uptake. Therefore, there is an important need to increase awareness of HZ and HZ vaccination during the pandemic.

**Key messages:**

• There is a need to increase awareness of HZ and HZ vaccination during the COVID-19 era.

• Further studies are needed to fully understand the impact of COVID-19 on the risk of HZ.

